# Computed tomographic Characteristics of Presumed Normal Canine Iliosacral Lymph Nodes

**DOI:** 10.1111/vru.70259

**Published:** 2026-09-23

**Authors:** Mohammad Momin Iqbal, Monique Mayer, Amy Larkin, Sarah Parker, Sally Sukut

**Affiliations:** ^1^ Department of Small Animal Clinical Sciences Western College of Veterinary Medicine University of Saskatchewan Saskatoon Saskatchewan Canada; ^2^ Western College of Veterinary Medicine Centre for Applied Epidemiology University of Saskatchewan Saskatoon Saskatchewan Canada

**Keywords:** abdominal lymph nodes, parietal lymph nodes, tumor response evaluation

## Abstract

Computed tomographic (CT) evaluation of medial iliac (MILN), internal iliac (IILN), and sacral (SLN) lymph nodes (LNs) in dogs provides vital staging information in certain neoplastic conditions. This retrospective study aimed to determine minimum transverse diameter (MinTD) and maximum transverse diameter (MaxTD) values for presumed normal iliosacral LNs and assess their utility in evaluating therapeutic response using response evaluation criteria in solid tumors (RECIST) v1.1. LN margins, number, and pre‐contrast attenuation were also analyzed. The CT scans of 71 dogs were reviewed by an ACVR‐certified veterinary radiologist. The MILNs were identified in 99% of dogs, predominantly bilaterally. The IILNs and SLNs were identified in 79% and 41% of dogs, respectively, with more variability in bilateral versus unilateral presence. Median values for MinTD/MaxTD were 4.2/7.4 mm for MILNs, 2.5/4.0 mm for IILNs, and 2.9/4.9 mm for SLNs. MinTD was <10 mm in all but one LN; MaxTD exceeded 10 mm in 19% of MILN sites and 1% of IILN sites. These results suggest that MinTD better reflects the RECIST v1.1 definition of non‐pathologic LNs (i.e., LNs <10 mm) and is the recommended measurement when evaluating therapeutic response. Transverse diameters, except MILN MaxTD, were larger in intact dogs (*p* ≤ 0.013), and females had larger SLN MaxTD (*p* = 0.047). Aortic diameter and body weight were positively correlated (*r* = 0.7, *p* < 0.001), and dogs with an aortic diameter >8.6 mm had greater diameters for MILNs and IILNs (*p* ≤ 0.034). Separate MinTD values for IILNs and SLNs are presented herein, whereas prior studies reported them as a combined group.

## 1 Introduction

The iliosacral lymphocenter, part of the parietal abdominal lymph nodes (LNs), includes the medial iliac (MILN), internal iliac (IILN), and sacral lymph nodes (SLN) [1, 2]. These LNs receive lymph from the dorsal abdominal skin, perineal and pelvic regions (including the tail base), abdominal wall muscles, and various pelvic, hip, and pelvic limb tissues (e.g., skin, fascia, muscles, joints, and bones). They also receive lymphatic drainage from parts of the urinary and caudal digestive tracts, peritoneum, genitalia, reproductive organs, and other LNs (superficial/deep inguinal, popliteal, medial femoral, and left colic); efferent channels drain into or merge with efferent vessels of the lumbar aortic LNs to form the pelvic lymphatic trunk [3, 4]. Metastatic involvement of the iliosacral LNs can occur with various neoplasms, including (but not limited to) prostatic carcinoma, apocrine gland anal sac adenocarcinoma, urothelial carcinoma of the urinary bladder, and multicentric lymphoma [5, 6, 7, 8, 9, 10]. In these patients, LN assessment on diagnostic imaging provides clinically useful information that guides staging and tissue sampling, treatment planning, prognostication, and evaluation of treatment response.

Computed tomography (CT) is the preferred imaging modality for assessing the iliosacral LNs owing to the increased sensitivity of CT for LN detection compared to ultrasound, which can be crucial for decision‐making regarding LN extirpation [1, 6]. Although several CT characteristics of iliosacral LNs have been reported, including size, shape, pre‐ and post‐contrast attenuation, and number [11], LN enlargement remains the most reliable indicator of malignancy [12], and serial transverse diameter measurements are commonly used to evaluate treatment response in both human and canine cancer patients [12, 13]. Previous reports on iliosacral LN size have been limited by the small sample sizes, inability to differentiate between IILNs and SLNs, and variability in reported size parameters and measurement methods [1, 11]. The human response evaluation criteria in solid tumors (RECIST v1.1) guidelines recommend using the “short axial” or minimal transverse diameter of a LN instead of the “longest diameter” [12]. However, separate measurements for the minimal transverse diameter (i.e., width or height, whichever is smallest) of presumed normal IILNs and SLNs have not been reported for dogs.

The primary objective of this study was to determine the minimum and maximum transverse diameter (MaxTD) values for presumed normal iliosacral LNs in dogs, while also describing margins, number, and pre‐contrast attenuation of the LNs. A secondary objective was to analyze the effect of age, gender, neuter status, LN laterality, and patient size determinants (body weight vs. aortic diameter) on LN transverse diameters.

## 2 Materials and Methods

### 2.1 Selection and Description of Subjects

A descriptive, retrospective study was conducted at the Western College of Veterinary Medicine (WCVM). Dogs that underwent pre‐contrast CT of the caudal abdomen and pelvic canal for any indication (soft‐tissue window, ≤5 mm slice thickness) between 2008 and 2018 were identified by searching electronic medical records and the picture archiving and communication system (PACS). The electronic medical records and CT scans of included dogs were retrospectively reviewed by an ACVR‐certified veterinary radiologist (S.S.) and a final‐year diagnostic imaging resident (A.L.), and patients with any known injury, neoplastic or other inflammatory disease process affecting a region draining to the iliosacral LNs, or those with multicentric malignancy (such as lymphoma), were excluded from the study. Dogs with congenital urinary anomalies were also excluded. In some CT studies, the field of view was collimated just caudal to the L7‐S1 junction; however, these cases were not excluded. This retrospective study used existing clinical records only. No animals were prospectively enrolled, no procedures or interventions were performed for research purposes, and no changes to clinical management occurred as part of the study. Owner and patient‐identifying information was not reported. In accordance with institutional policy, this study was exempt from animal ethics approval, and an animal use protocol was not required.

### 2.2 Clinical Data

The electronic medical records were reviewed by a final‐year diagnostic imaging resident (A.L.), and the age, weight, breed, gender, and neuter status were recorded for all dogs. The reasons for imaging and final diagnosis (when available) were also recorded. If a final diagnosis was not available, the imaging diagnosis from the radiology report was recorded.

### 2.3 CT Image Acquisition Protocol

CT scans were performed at WCVM using a 16‐slice multi‐detector CT scanner (Aquilion 16, Toshiba America Medical Systems, USA) prior to November 2017, whereas all subsequent scans utilized a 320‐detector row CT scanner (Aquilion One Vision, Toshiba America Medical Systems, USA). Images were acquired in helical scan mode (pitch factor 0.656, matrix size 512 cm × 512 cm, tube rotation time 0.5 s) and reconstructed using a soft‐tissue algorithm. Other technical parameters varied, including mAs (100–300), kVp (100–120), slice thickness (0.5–5 mm), and field‐of‐view, which was tailored individually. Patients were sedated or anesthetized and scanned in either dorsal or sternal recumbency with the pelvic limbs extended.

### 2.4 Image Evaluation

**FIGURE 1:**
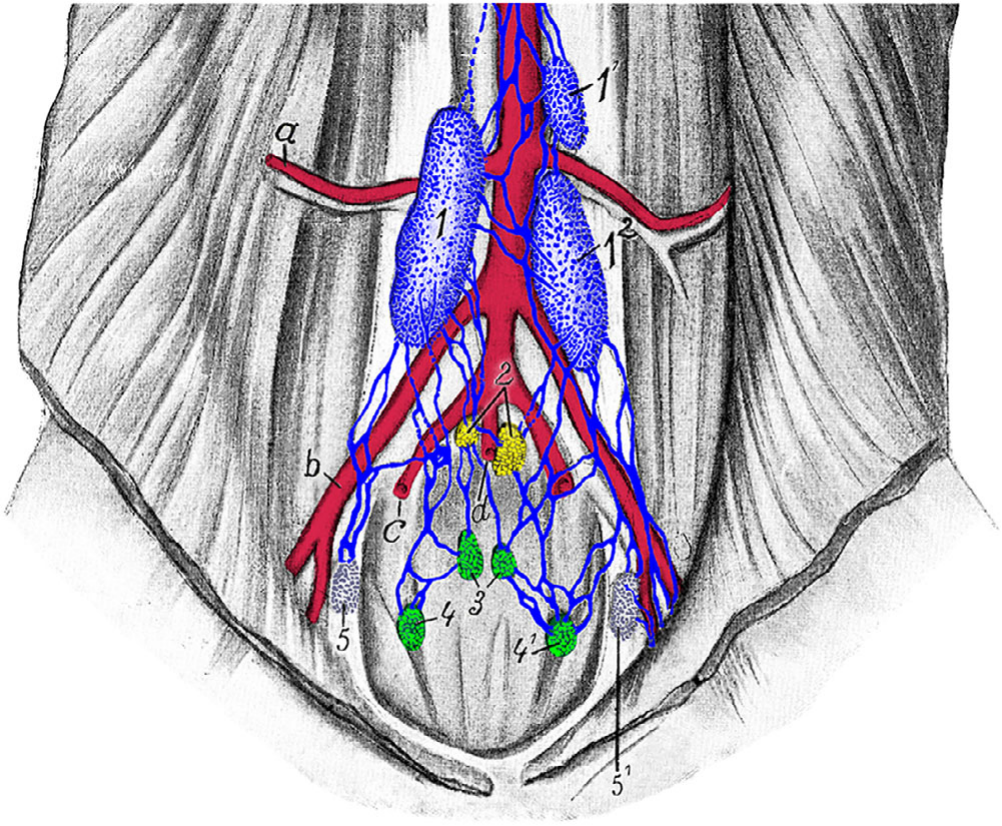
The iliosacral lymph nodes of the dog. a, Right deep circumflex iliac artery; b, right external iliac artery; c, right internal iliac artery; d, median sacral artery. 1, 1^1^, 1^2^ Medial iliac lymph nodes (blue); 2, internal iliac lymph nodes (yellow); 3, medial sacral lymph nodes (green); 4, 4^1^, lateral sacral lymph nodes (green); 5, 5^1^, deep inguinal lymph nodes (purple) (not considered part of the iliosacral lymph node group). The medial and lateral sacral lymph nodes were considered a single group in the current study. Permission: Courtesy of Monique Mayer, Department of Small Animal Clinical Sciences, Western College of Veterinary Medicine, University of Saskatchewan, based on https://www.saskoer.ca/k9lymphaticsystem/chapter/figure‐27‐lymph‐vessels‐of‐the‐kidneys‐and‐lymph‐nodes‐along‐the‐abdominal‐aorta‐and‐its‐terminal‐branches/.

**FIGURE 2:**
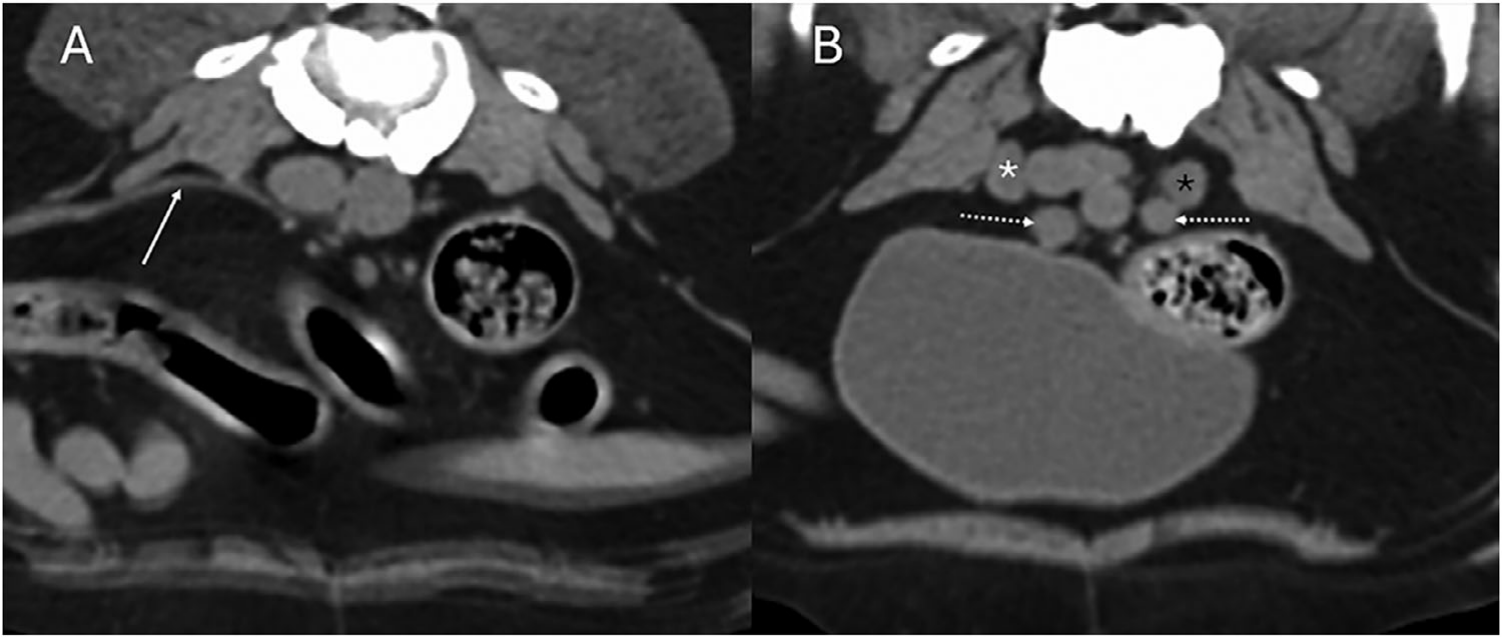
Pre‐contrast transverse CT images of the caudal abdomen (A and B) from a single patient, viewed in a soft‐tissue window (window level = 40 HU, window width = 400 HU) with 2 mm slice thickness and acquired in sternal recumbency. The left side of the dog is on the right side of the images. (A) Transverse slice at the level of the L5–6 intervertebral disc space. The solid white arrow indicates the right deep circumflex iliac artery. (B) At the level of the L6 caudal end plate, the external iliac arteries are indicated by dotted arrows. The right MILN (white asterisk) is noted between the right deep circumflex iliac artery seen in (A) and the ipsilateral external iliac artery. Note a left‐sided MILN in the same image (black asterisk).

**FIGURE 3:**
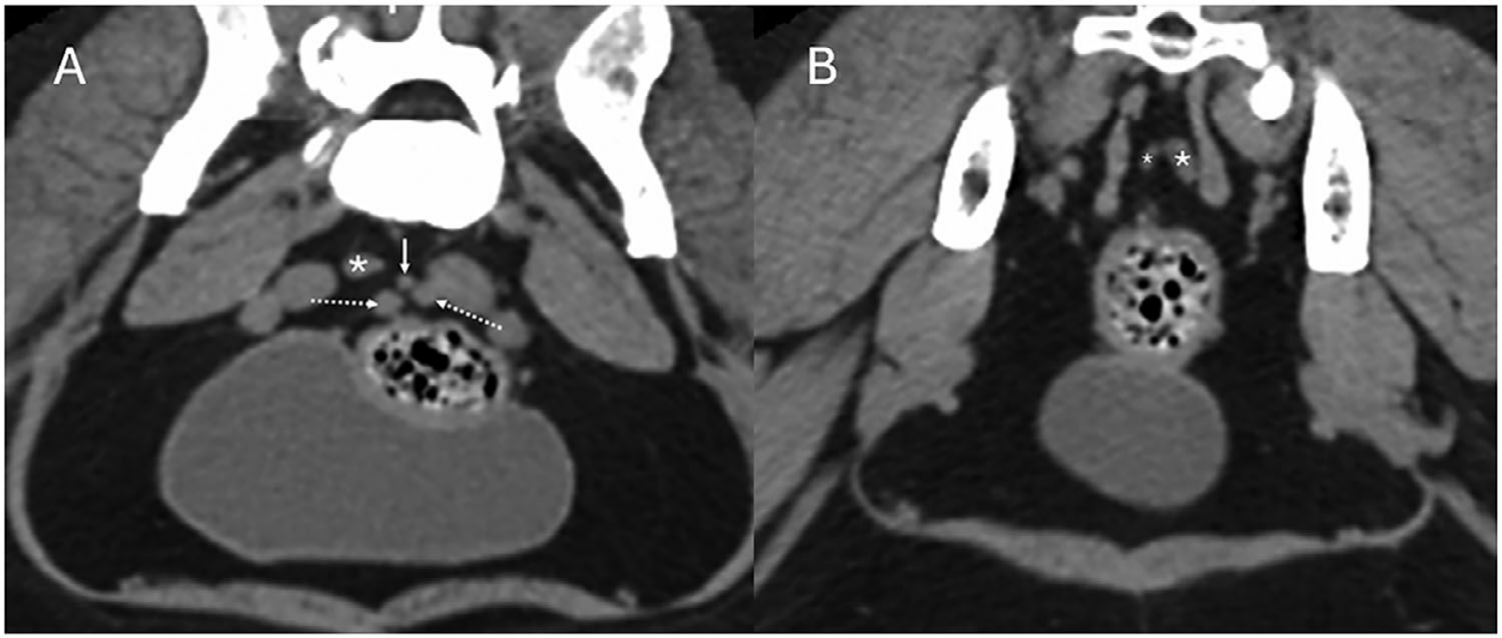
Pre‐contrast transverse CT images of the caudal abdomen (A and B) from the same patient in Figure 2. Images are viewed in a soft‐tissue window (window level = 40 HU, window width = 400 HU) with 2 mm slice thickness and acquired in sternal recumbency. The left side of the dog is on the right side of the images. (A) Slice acquired at the caudal end plate of L7. The internal iliac arteries (dotted arrows) and median sacral artery (solid arrow) are highlighted. In the angle formed between the ipsilateral internal iliac artery and median sacral artery, the IILN (*) is identified. (B) The median sacral artery from (A) is followed caudally to the level of the sacrum, where the right and left SLNs (*) are identified.

Transverse pre‐contrast images were evaluated by an ACVR‐certified veterinary radiologist (S.S.) in soft tissue window (width 400 Hounsfield Units (HU); level 40 HU) using DICOM image evaluation software (McKesson Radiology Station version 12.2, McKesson, San Francisco, CA). LNs were identified on the basis of their anatomical location relative to surrounding structures, as previously described [3], and depicted in Figure 1. MILNs were defined as LNs located adjacent to the aorta (left) and caudal vena cava (right), ventral to the fifth and sixth lumbar vertebral bodies, and bordered cranially and caudally by the deep circumflex iliac and external iliac arteries, respectively [3], as illustrated in Figure 2A,B. LNs positioned just caudal to the MILNs, between the internal iliac and median sacral arteries (level of the sixth and seventh lumbar vertebrae), were classified as IILNs [3], as shown in Figure 3A. The LNs positioned further caudal to the IILNs, ventral to the sacrum and immediately adjacent to the median sacral artery, were classified as the SLNs [3], as depicted in Figure 3B. LNs were evaluated using post‐contrast sequences when available, which provided improved differentiation from adjacent vessels and soft tissue structures. Structures smaller than 1 mm, particularly those with uncertain nodal origin, were not recorded due to the difficulty in confidently distinguishing them as LNs on CT.

**FIGURE 4:**
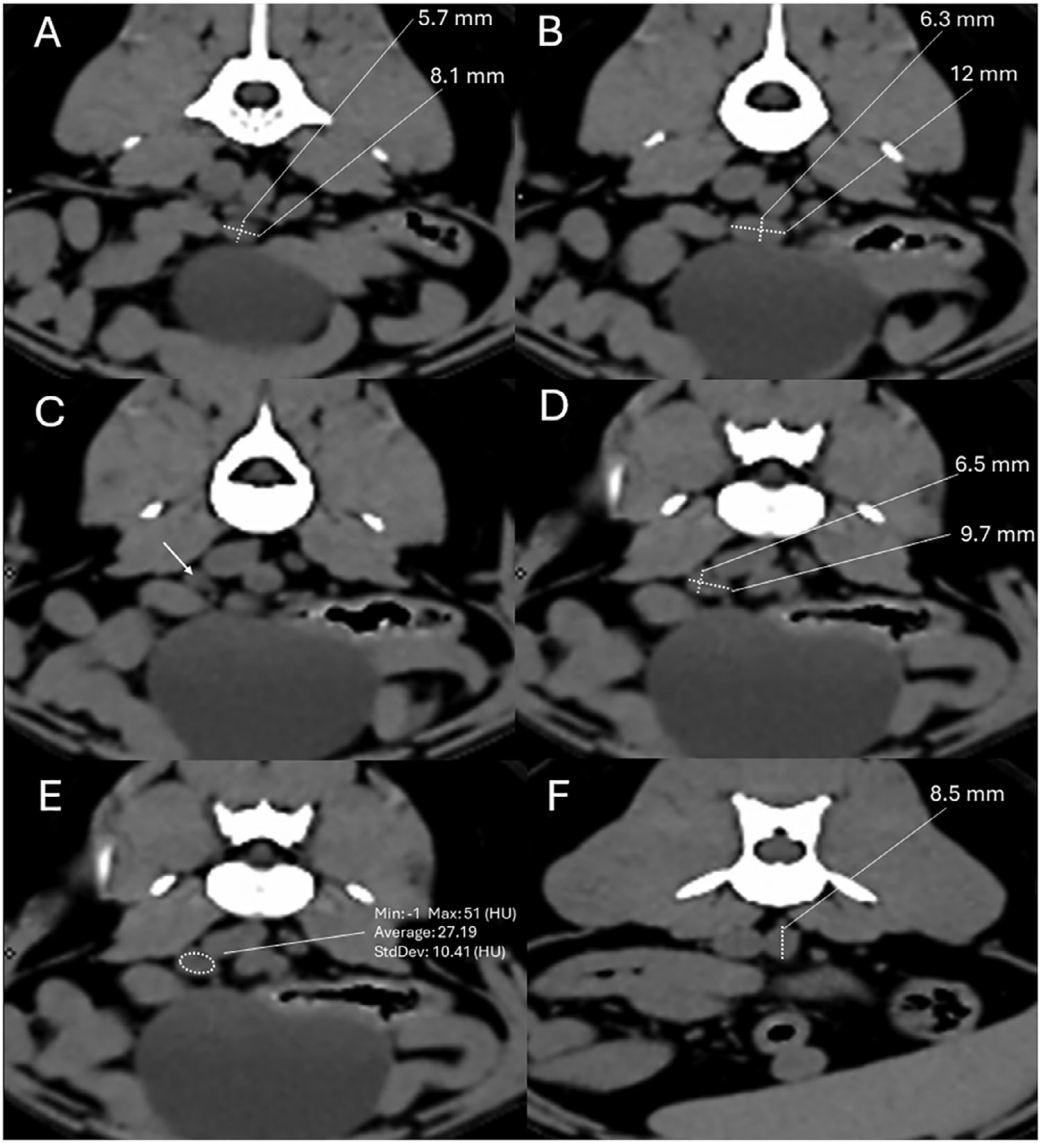
Pre‐contrast transverse CT images of the MILNs (A–E) and abdominal aorta (F) from a single patient. All images are viewed in a soft‐tissue window (window level = 40 HU, window width = 400 HU) with 1 mm slice thickness and acquired in dorsal recumbency. The left side of the dog is on the right side of the images. The dotted white lines represent MinTD and MaxTD measurements, respectively. (A) The right MILN measures 5.7 mm (MinTD) × 8.1 mm (MaxTD). (B) The same LN measured in (A) is viewed in another, slightly caudal slice, now measuring 6.3 mm (MinTD) × 12 mm (MaxTD). (C and D) The solid white arrow indicates a second MILN on the right side, viewed across two different slices, and measures 6.5 mm (MinTD) × 9.7 mm (MaxTD). After considering both MILNs on the right side across all viewed slices, the maximum values obtained for the MinTD and MaxTD are 6.5 mm (D) and 12 mm (B), respectively. Note that these MinTD and MaxTD values are obtained from two different MILNs on the right side and are not measured on the same slice. (E) Pre‐contrast attenuation is measured by placing the largest possible elliptical region of interest (dotted white circle) at the LN from the same slice in (D) where the maximum value for MinTD was recorded, while avoiding the LN margins. A mean pre‐contrast attenuation value of 27.19 HU is recorded. (F) Dashed white line indicating the maximum dorsoventral diameter of the aorta (8.5 mm), measured at the L5 cranial end plate.

Each side (left vs. right) in a dog where at least one LN from one of the three LN groups (MILN, IILN, or SLN) could be identified was considered one site. In each dog, the number of LNs identified per site was recorded. For all identified LN sites, the MaxTD and minimum transverse diameter (MinTD) were recorded in millimeters, and pre‐contrast attenuation was measured in HU. The MaxTD was defined as the greatest nodal dimension in the transverse plane, that is, the height or width, whichever was largest. The second largest transverse dimension, perpendicular to the MaxTD, was defined as the MinTD [12, 14, 15]. Transverse diameters were measured using transverse slices [11]. If more than one LN was present at a site, the node with the largest transverse diameter was used for the measurement [15]. Using this method, the MaxTD and MinTD were separately assessed and possibly measured on different slices or on different LNs at a particular site [15], as illustrated in Figure 4A–D. The MinTD slice was then used to obtain the minimum, maximum, and mean pre‐contrast attenuation values for the LN by placing the largest possible region of interest (ROI) while avoiding the LN margins (Figure 4E). The outer LN margins on this same MinTD slice were assessed, and the LN was classified as either well‐defined (clearly delineated from surrounding tissues) or ill‐defined (poor delineation from surrounding tissues) [15]. As both veterinary and human literature recommend using only transverse diameters for cancer therapy response evaluation, LN length was not evaluated in this study [12, 13]. Furthermore, the reformatted images used in measuring LN length may introduce inaccuracy [15]. To analyze the relationship between body size and LN size, aortic luminal diameter was included as an indicator of patient size, as it is less influenced by body habitus. The maximum transverse dorsoventral diameter of the aorta was measured (in millimeters) at the level of the cranial end plate of the fifth lumbar vertebral body (Figure 4F). If a vertebral anomaly was noted, vertebrae were counted cranially from the sacrum to locate L5.

### 2.5 Statistical Analysis

LN transverse diameter and attenuation measurements were assessed for normality using the Shapiro–Wilk test and visually with stem‐and‐leaf plots. For nonparametric data, descriptive statistics (median, fifth, and 95th percentiles) were reported for LN diameter and attenuation.

Initially, univariable analysis (multilevel linear regression) was conducted to identify variables that were potentially associated (*p* < 0.20) with LN transverse diameter. The following variables were considered: neuter status (intact vs. neutered), weight (≤13.7 vs. >13.7 kg), transverse aortic diameter (≤8.6 vs. >8.6 mm), laterality (left vs. right side), gender (female vs. male), and age (≤71.5 vs. >71.5 months). The categories for the continuous variables (weight, transverse aortic diameter, and age) were created on the basis of their respective median values. Collinearity between predictor variables was evaluated with Pearson correlation for continuous variables and with frequency tables for categorical variables. As multiple LNs were included from the same dog, the effect of clustering by dog was controlled for by including the dog as a random effect. Backward stepwise, multilevel, and multivariable regression analysis was then performed by retaining variables considered to be important risk factors (*p* < 0.05). Confounders were retained in the final model if removing the variable changed the coefficient of significant predictors by more than 20%. Significant variables were also evaluated for the presence of interactions.

A single author (M.M.) performed all statistical tests using commercial statistical software (STATA/SE version 16.0 for Windows, StataCorp, College Station, TX, USA), with guidance from an analytical epidemiologist (S.P.).

## 3 Results

### 3.1 Clinical Findings

A total of 198 dogs met the inclusion criteria. Following application of exclusion criteria, 71 dogs were enrolled in the study, comprising 36 females (30 spayed, 6 intact) and 35 males (31 castrated, 4 intact). There were 47 purebred dogs, comprising 27 different breeds, including Alaskan Malamute (*n* = 1), American Cocker Spaniel (*n* = 2), American Pitbull (*n* = 1), Beagle (*n* = 8), Bichon Frise (*n* = 2), Border Collie (*n* = 1), Boston Terrier (*n* = 1), Boxer (*n* = 2), Chihuahua (*n* = 2), Coonhound (*n* = 1), Dachshund (*n* = 2), Doberman Pinscher (*n* = 1), German Shepherd (*n* = 2), Golden Retriever (*n* = 3), Great Dane (*n* = 1), Irish Wolfhound (*n* = 1), Labrador Retriever (*n* = 2), Maltese (*n* = 1), Miniature Dachshund (*n* = 2), Old English Sheepdog (*n* = 1), Pug (*n* = 2), Rottweiler (*n* = 1), Shih Tzu (*n* = 1), Toy Poodle (*n* = 1), Weimaraner (*n* = 1), Whippet (*n* = 1), and Yorkshire Terrier (*n* = 3). The remaining 24 dogs were mixed breed. Median age of the canine population was 72 months (range: 4–175 months), median weight was 13.7 kg (range: 1.4–56 kg), and median aortic diameter was 8.6 mm (range: 4.3–15 mm).

Forty‐five percent (32/71) of included dogs had normal abdominal CT studies based on the absence of imaging abnormalities. In these 32 dogs, CT studies were performed for the following reasons: for an unrelated nutrition study (*n* = 14); oncological staging for non‐abdominal (head, neck, forelimb, and rib) neoplasms (*n* = 8); extra‐cranial work‐up for idiopathic epilepsy (*n* = 4); work‐up for megaesophagus and/or aspiration pneumonia (*n* = 4); and work‐up for urinary incontinence (presumed final diagnosis of urethral sphincter mechanism incompetency, negative for cystitis and infection on urine sediment analysis and culture) (*n* = 2). Of the remaining dogs with abnormal findings on CT imaging, 14% (10/71) dogs had CT findings consistent with intra‐ or extrahepatic portosystemic shunts (surgically confirmed in 4 of the dogs); 10% (7/71) dogs had CT findings, histopathological or cytological diagnoses consistent with hepatocellular carcinoma; 9% (6/71) dogs had CT findings consistent with an adrenal mass; 7% (5/71) dogs had gastrointestinal foreign bodies and/or imaging features consistent with gastroenteritis; 4% (3/71) dogs had imaging features consistent with biliary tract obstruction; 3% (2/71) dogs had normal caudal abdominal/pelvic CT with histopathologically confirmed pelvic limb lipomas; and 3% (2/71) had imaging features consistent with degenerative lumbosacral disease. Intervertebral disc protrusion, pulmonary mass (final histopathologic diagnosis of adenocarcinoma), pancreatic nodule (final histopathologic diagnosis of islet cell carcinoma), and diaphragmatic hernia (presumed congenital) represented 1% (1/71) of the study population each.

### 3.2 CT Findings

Each left and right side of every dog was considered a separate anatomical site. Accordingly, 142 potential MILN sites (71 dogs × 2 sides) were evaluated. MILNs were identified at 134 of these sites in 70 of 71 dogs (99%). They were identified bilaterally in 64 dogs, only on the left side in 5 dogs, only on the right side in 1 dog, and were not identified on either side in 1 dog. Similarly, 142 potential IILN sites were evaluated. IILNs were identified at 84 of these sites in 56 of 71 dogs (79%). They were identified bilaterally in 28 dogs, only on the left side in 5 dogs, only on the right side in 23 dogs, and were not identified on either side in 15 dogs. In 12 dogs, the sacrum was not included in the CT scan; therefore, only 59 dogs (118 potential sites) could be evaluated for SLNs. SLNs were identified at 34 of these sites in 24 of 59 dogs (41%). They were identified bilaterally in 10 dogs, only on the left side in 10 dogs, only on the right side in 4 dogs, and were not identified on either side in 35 dogs.

The number of LNs per site and their margins at each site are summarized in Table 1. At most sites, a single LN was identified, and the majority of sites had well‐defined LNs. Descriptive statistics for transverse diameter and pre‐contrast attenuation are summarized in Table 2. On the basis of lack of normality, the median, fifth, and 95th percentile values are presented. The MinTD was ≥10 mm at <1% (1/134) of the examined MILN sites, and in 0% of the examined IILN (0/84) and SLN (0/34) sites. In the single dog where the MinTD exceeded 10 mm, the MILN on the right side had a MinTD of 13 mm. The MinTD was ≥5 mm in 35% (47/134) of the MILN sites and in <3% each of the examined IILN (2/84) and SLN (1/34) sites. The MaxTD was ≥10 mm at 19% (26/134) of the examined MILN sites, 1% (1/84) of the IILN sites, and 0% (0/34) of the SLN sites. The MaxTD was ≥5 mm in approximately 81% (109/134) of the MILN sites, 36% (30/84) of the IILN sites, and 47% (16/34) of the SLN sites.

**TABLE 1:** Descriptive statistics for lymph node number and margins of presumed normal medial iliac lymph nodes (MILNs) and internal iliac lymph nodes (IILNs) in 71 dogs, and sacral lymph nodes (SLNs) in 59 dogs.

	MILN	IILN	SLN
Total number of sites^a^ (*n*)	134	84	34
Number of lymph nodes per site^a^			
1	86/134 (64%)	74/84 (88%)	34/34 (100%)
2	40/134 (30%)	10/84 (12%)	0/34 (0%)
3	8/134 (6%)	0/84 (0%)	0/34 (0%)
Lymph node margination			
Well‐defined	116/134 (87%)	73/84 (87%)	22/34 (65%)
Ill‐defined	18/134 (13%)	11/84 (13%)	12/34 (35%)

^a^ A site refers to the left or right side of a dog where an MILN, IILN, or SLN is identified, with each side considered a separate site for each lymph node type.

**TABLE 2:** Descriptive statistics for transverse diameter and attenuation of presumed normal medial iliac lymph nodes (MILNs) and internal iliac lymph nodes (IILNs) in 71 dogs, and sacral lymph nodes (SLNs) in 59 dogs.

	MinTD (mm)	MaxTD (mm)	Attenuation (HU)
MILN (*n* = 134)	4.2 (2.0–7.8)	7.4 (3.4–13.0)	33.15 (13.47–57.34)
IILN (*n* = 84)	2.5 (1.4–4.5)	4.0 (1.8–8.5)	31.16 (2.92–49.62)
SLN (*n* = 34)	2.9 (1.4–4.4)	4.9 (2.0–9.2)	29.44 (0.50–47.06)

*Note*: On the basis of lack of normality, median, fifth to 95th percentiles are presented.

Abbreviations: HU, Hounsfield Units; MaxTD, maximum transverse diameter; MinTD, minimum transverse diameter.

Aortic diameter and body weight were strongly correlated (*r* = 0.7, *p* < 0.001) and deemed to be biologically reflecting the same parameter of patient size. Hence, only aortic diameter was used in multivariable analysis (Table 3). Age category was not associated with transverse diameters for any of the LNs (*p* ≥ 0.223), hence not considered further in multivariable analysis. Dogs with an aortic diameter >8.6 mm had greater transverse diameters for MILNs and IILNs (*p* ≤ 0.034), whereas no significant association was noted between aortic diameter category and SLN transverse diameters (*p* ≥ 0.526). Intact dogs had greater transverse diameter measurements for all LNs (*p* ≤ 0.013), except for MaxTD of MILNs, which was not associated with neuter status (*p* = 0.157). Gender was not significantly associated with transverse diameters (*p* ≥ 0.101), except for the MaxTD of SLNs, which was greater in female dogs than in male dogs (*p* = 0.047). The MILNs on the right side had significantly greater MinTD than those on the left (*p* = 0.013), whereas no association between LN laterality and transverse diameters was noted in any of the remaining iliosacral LNs (*p* ≥ 0.105). For all models evaluated, no significant interaction terms were detected.

**TABLE 3:** Variables associated with computed tomographic (CT) transverse diameter of medial iliac lymph nodes (MILNs) and internal iliac lymph nodes (IILNs) in 71 dogs, and sacral lymph nodes (SLNs) in 59 dogs.

		Prediction coefficient	95% CI	*p* value
MILN				
	MinTD			
	Neuter status (intact > neutered)	1.5	0.4–2.5	0.006
	Side (right > left)	0.4	0.1–0.7	0.013
	Aortic diameter category (>8.6 mm) > (≤8.6 mm)	1.6	0.9–2.4	<0.001
	MaxTD			
	Aortic diameter category (>8.6 mm) > (≤8.6 mm)	2.9	1.8–4.0	<0.001
IILN				
	MinTD			
	Neuter status (intact > neutered)	1.1	0.5–1.7	<0.001
	Aortic diameter category (>8.6 mm) > (≤8.6 mm)	0.5	0.0–0.9	0.034
	MaxTD			
	Neuter status (intact > neutered)	1.6	0.4–2.9	0.011
	Aortic diameter category (>8.6 mm) > (≤8.6 mm)	1.3	0.4–2.2	0.005
SLN				
	MinTD			
	Neuter status (intact > neutered)	1.0	0.2–1.8	0.013
	MaxTD			
	Neuter status (intact > neutered)	2.1	0.5–3.6	0.011
	Gender (female > male)	1.2	0.0–2.4	0.047

*Note*: Statistically significant prediction coefficients for lymph node transverse diameters for the analyzed variables following multilevel, multivariable analysis. Variables were retained if significant predictors (*p* ≤ 0.05) or important confounders.

Abbreviations: CI, confidence interval; MaxTD, maximum transverse diameter; MinTD, minimum transverse diameter.

## 4 Discussion

The detection frequency of MILNs has varied in previous CT and ultrasound studies, with some detecting 100% of the right and left MILNs (i.e., at least one LN on each side) [11, 16, 17]. The 99% detection rate in the present study aligns with these findings and with anatomical literature describing MILNs as consistently present [4, 11, 17]. Unilateral MILNs were, however, observed in some dogs, and one dog had no detectable MILNs. Asymmetric detection of MILN presence has been reported in ultrasound studies, with the right MILN seen in 82% and the left in 45% of dogs [18]. Discrepancies in LN detection on ultrasound may result from its lower spatial resolution, potentially limiting visibility or leading to misidentification of IILNs as MILNs due to their proximity. Additional limitations, particularly when scanning in dorsal recumbency using a ventral approach, include acoustic interference from colonic gas and increased distance between the transducer and LNs, especially with urinary bladder distension [18]. On CT, the detection of LNs may be limited by both patient‐related and technical factors. Reduced caudal abdominal and intra‐pelvic fat can hamper differentiation of LNs from adjacent soft tissues [11]. Insufficient LN size (i.e., below the scanner's spatial resolution) can result in ill‐defined or undetectable LNs due to partial volume averaging, and a prominent fat‐attenuating hilus in relatively small LNs can impact the LN's attenuation and hinder definitive identification. The lack of post‐contrast sequences in some cases can restrict differentiation of LNs from surrounding soft tissue and vasculature, and thicker CT slices can also reduce detection. Although one canine study reported no impact of slice thickness on LN detection [11], a later feline study [19] showed significantly improved detection of intra‐thoracic LNs with thinner slices (0.625 and 1.25 mm) compared to 2.5 and 5.0 mm slices (*p* ≤ 0.0109) and noted clearer visualization only on post‐contrast images. Taken together, these factors likely explain the unilateral detection of MILNs in some dogs and the complete lack of MILN visualization in one case in the present study.

The IILN detection rate (79%) in the present study is consistent with previous reports of 83% (25/30) [1] and 74% (14/19) for IILNs and SLNs combined [11]. However, SLNs were only detected in 41% of dogs in the present study, possibly because these LNs are only present in about half the canine population [3]. This number is slightly lower than the previously reported 57% (17/30) detection rate [1]. Because IILNs and SLNs are typically smaller than MILNs [4], their detection is likely similarly limited by slice thickness as well as the other CT‐related limitations noted above. The laterality distribution for IILNs and SLNs could not be compared with prior studies, as the only other study reporting laterality could not distinguish between these two LN groups [11].

An increased number of MILNs detectable on imaging has been linked to malignancy in a previous ultrasound study [18]. However, a later CT study reported the presence of more than two MILNs in 21% of dogs [11], which is consistent with the results of the present study, which identified two nodes at 30% and three nodes at 6% of sites. The median pre‐contrast attenuation values of the iliosacral LNs in the present study (Table 2) are consistent with the reported 37 HU (range 20–52 HU) for normal abdominal LNs in dogs [11] and are also comparable to values reported in another study of abdominal LNs, in which MILNs demonstrated similar pre‐contrast attenuation ranges [17]. Pre‐contrast attenuation uniformity or post‐contrast enhancement patterns were not evaluated in this study for the following reasons: (a) inability to control certain technical parameters, such as kVp, which can influence attenuation values; (b) substantial variability in post‐contrast attenuation values resulting from inconsistent timing between contrast administration and image acquisition [11], a limitation of retrospective study designs; and (c) absence of post‐contrast sequences in a portion of cases. Wide variability in pre‐ and post‐contrast enhancement patterns of LNs has been reported across studies, with overlap between normal and pathological nodes, making imaging‐based differentiation challenging. This overlap is evident in prior studies describing pre‐contrast peripheral hyperattenuation and mild heterogeneity in normal abdominal and sternal LNs, absence of post‐contrast enhancement in both normal and pathologic tracheobronchial LNs, and peripheral enhancement in both malignant tracheobronchial and presumed normal abdominal LNs [11, 20, 21]. Exploring the significance of these characteristics across normal and pathologic LNs requires further research, including comparisons with histopathological diagnoses and controlled technical parameters.

Varying size parameters for normal canine iliosacral LNs have been reported in the veterinary literature. In general, the transverse diameter measurements reported herein were consistent with previous reports. One study evaluating abdominal LNs in 19 dogs reported the length (maximal LN dimension), width (second maximal dimension, in transverse images), and thickness (the third transverse dimension, perpendicular to the width) of iliosacral LNs [11]. Their reported mean dimensions (length x width x thickness) were 22.8 mm × 6.7 mm × 4.6 mm for the MILNs. That study was unable to differentiate between IILNs and SLNs but reported the combined mean dimensions of 10.3 mm × 4.8 mm × 3.7 mm for these two LNs. Despite the smaller sample size of that report, the MaxTD and MinTD values in the present study were consistent with their width and thickness, respectively. Another study evaluating 30 presumed normal dogs reported only the mean maximum diameters (largest transverse dimension) for the MILNs (7.4 mm), IILNs (5.5 mm), and SLNs (4.8 mm) [1]. The MaxTD values in the present study were consistent with these findings. Direct comparison of transverse nodal dimensions with a CT study evaluating multiple abdominal LNs, including the MILNs, in 45 dogs was not possible because the authors reported nodal length, thickness, and width without specifying which transverse dimension represented the maximum versus MinTD [17]. Subsequent studies assessing iliosacral lymphadenomegaly have used varying subjective and objective criteria, in addition to varying measurement methods while assessing LNs, including comparison of the dorsoventral height of the LN to the internal iliac artery or comparison to the contralateral LN [6, 22, 23]. In another study, LN thickness exceeding 5 mm was considered suggestive of metastatic involvement; however, the method used to measure nodal thickness was not described [10]. The findings of the present study do not support a 5 mm cutoff, as both transverse diameters (MinTD and MaxTD) frequently exceeded this threshold in the MILNs; MinTD was ≥5 mm in 35% of sites and MaxTD in 81% of sites. Conversely, MinTD remained < 10 mm in all but one examined LN site (likely an outlier), supporting the RECIST v1.1 criterion that LNs with a short axis diameter less than 10 mm are considered normal or indicative of a complete response (CR) [12]. The authors recognize the importance of using well‐described, consistent measurement methods when assessing these LNs. Although establishing criteria for metastatic LN enlargement was not the focus of this study and would require histopathologic confirmation, the authors propose that MinTD values exceeding 10 mm may warrant increased clinical suspicion and further diagnostic investigation.

Human guidelines recommend using the short axis measurement (MinTD in this study) as it is more reliable and reproducible, being less affected by LN orientation on CT and a better predictor of malignancy [12]. In contrast, veterinary guidelines recommend using the longest diameter (MaxTD in this study) for serial assessments [13]. Findings of the present study suggest that MinTD (which was <10 mm in all but one examined LN site) appears appropriate for defining non‐pathologic iliosacral LNs on CT, consistent with RECIST v1.1 criteria [12]. In contrast, MaxTD frequently exceeded 10 mm, similar to findings from a recent study evaluating presumed normal cervical LNs in dogs [15]. These findings also suggest that MinTD, as recommended by human literature, may be more applicable to tumor response evaluation than MaxTD. Interestingly, results from the present study also indicate that the MinTD is most consistent with MILN dimensions reported in the anatomical literature [4]. The published anatomical measurements for MILNs are length × width × thickness = 40.0 mm × 20.0 mm × 5.0 mm [4]. Although a prior CT study [11] reported their MILN measurements to be smaller than these anatomical values, both the median MinTD from the present study (4.2 mm) and the reported mean thickness from that CT study (4.6 mm) closely align with the anatomical reference thickness of 5 mm. In contrast, the median MaxTD from the present study (7.4 mm) and the mean width reported in the aforementioned CT study (6.7 mm) are not consistent with the anatomic reference width of 20 mm.

In the present study, greater transverse LN diameters were observed in intact dogs. The association between neuter status and iliosacral LN size has not previously been investigated. The exact cause of the observed size discrepancy remains unknown, and this association warrants further investigation, including cytologic or histologic sampling. MILNs on the right side were, on average, 0.4 mm larger in MinTD than those on the left, contrary to a previous ultrasound study, which found no difference in LN size between the right and left sides [16]. Additionally, the MaxTD for SLNs was greater in females. Although statistically significant, as highlighted in Table 3, these small size differences based on neuter status, laterality, and gender are likely not clinically meaningful.

This study also aimed to analyze the association between patient size and LN size, given that LN size is expected to increase with patient size [16, 17]. Consistent with a prior study [24], the observed collinearity between aortic diameter and body weight in the present study suggests that aortic diameter, like body weight, serves as an indicator of patient size. However, in obese patients, higher body weight may lead to an overestimation of patient size. By using aortic luminal diameter instead of body weight in the statistical model, the association between patient size and LN size was analyzed while potentially minimizing the confounding effects of body habitus and the different body shapes and weights across multiple dog breeds evaluated. As expected, a statistically significant association was observed between aortic diameter category and transverse diameters of the MILNs and IILNs. However, no association was noted with SLN transverse diameters. Such an association may not exist, or the power of this study (with only 34 SLN sites) may have been insufficient for this assessment.

A previous study introduced a normalized ratio of the maximum iliosacral LN diameter (i.e., MaxTD) to aortic diameter to establish criteria for LN enlargement [1]. Although results from the present study support the use of aortic diameter for normalizing LN size, a normalized LN‐to‐aortic diameter ratio is not reported herein, nor are separate reference values provided based on aortic diameter, given the small mean differences observed in transverse diameters across the aortic diameter categories (see Table 3). The clinical significance of these small differences is questionable considering the limitations of CT spatial resolution in a retrospective study design. In routine clinical practice, LN evaluation considers the entire clinical picture rather than focusing on a single characteristic, such as enlargement. Enlarged LNs can be non‐pathologic, and normal‐sized LNs can be metastatic [25]. Furthermore, given the significant variation in LN size across different neoplastic etiologies [12] and even within stages of the same neoplastic process, using LN size alone or applying a specific cutoff value is not optimal for LN assessment. As no pathognomonic imaging features for metastatic LNs exist, diagnosis should ultimately rely on histopathologic or cytologic confirmation [26]. The RECIST guidelines use LN measurements solely for comparison with prior or future measurements of the same LN, making the measurement at a single point in time less relevant.

The present study has several limitations, mainly due to its retrospective design. CT scans were performed with varying slice thicknesses, occasionally up to 5 mm, which may have affected the visibility of smaller LNs. Additionally, post‐contrast sequences were not available for all dogs, potentially reducing LN detectability. Furthermore, post‐contrast enhancement patterns may be affected by the significant variability in timing between contrast administration and image acquisition. As a result, post‐contrast images were excluded from analysis, and enhancement characteristics could not be evaluated. Although the MinTD was recorded for each LN, the specific anatomical orientation of this measurement (i.e., dorsoventral vs. mediolateral) was not systematically documented and varied between LNs. This study lacked cytologic or histopathologic examination to confirm the normal status of LNs, which precluded further investigation of associations between LN size and variables like neuter status and gender. Additionally, four of the included dogs had aspiration pneumonia. A previous study reported intrathoracic lymphadenopathy in 26.3% of dogs with aspiration pneumonia [27]; however, the presence or absence of generalized LN involvement was not assessed in that study. Therefore, generalized LN reactivity or mild enlargement secondary to systemic inflammation could not be completely excluded in these dogs. Aortic diameter, which has been used in previous ultrasound and CT‐based studies to normalize the renal, left atrial, portal vein, and iliosacral LN sizes [1, 24, 28, 29], may fluctuate due to factors such as hydration status, cardiac cycle, or vascular conditions like systemic hypertension [24]. Most of these prior studies noted that such factors could not be controlled, and similar limitations applied to the present study.

In conclusion, transverse diameter values (MinTD and MaxTD) have been reported in the largest canine population evaluating the iliosacral lymphocenter on CT. Individual MinTD values for the IILNs and SLNs have not previously been reported in a presumed normal canine population. When evaluating therapeutic response in cancer patients undergoing treatment as per the RECIST v1.1 framework, the MinTD (i.e., width or height, whichever is smallest) is most consistent with the definition of non‐pathologic LNs or a CR, defined as a transverse diameter of less than 10 mm. This aligns with the human RECIST v1.1 guidelines, and future revisions of veterinary response evaluation criteria for solid tumors should consider adopting MinTD rather than the traditionally used MaxTD. Associations between LN size and gender, neuter status, and laterality are reported. Age was not associated with LN size, consistent with prior reports [16, 30]. Aortic diameter can serve as an indicator of patient size and is positively correlated with the transverse diameters of MILNs and IILNs.

## Author Contributions

Conception and design: Mohammad Momin Iqbal, Sally Sukut, Monique Mayer, Amy Larkin, and Sarah Parker. Acquisition of data: Mohammad Momin Iqbal, Sally Sukut, Amy Larkin, and Sarah Parker. Analysis and interpretation of data: Mohammad Momin Iqbal, Sally Sukut, Monique Mayer, Amy Larkin, and Sarah Parker. Drafting the article: Mohammad Momin Iqbal. Revising the article for intellectual content: Mohammad Momin Iqbal, Sally Sukut, Monique Mayer, Amy Larkin, and Sarah Parker. Final approval of the completed article: Mohammad Momin Iqbal, Sally Sukut, Monique Mayer, Amy Larkin, and Sarah Parker. Agreement to be accountable for all aspects of the work in ensuring that questions related to the accuracy or integrity of any part of the work are appropriately investigated and resolved: Mohammad Momin Iqbal, Sally Sukut, Monique Mayer, Amy Larkin, and Sarah Parker.

## Disclosure

The authors disclose that findings were not previously presented at a scientific meeting. This is an original unpublished manuscript, not under editorial consideration by another journal. No reporting checklist was used.

## Conflicts of Interest

The authors declare no conflicts of interest.

## Data Availability

The data that support the findings of this study are available from the corresponding author (M.M.I.) upon reasonable request.
